# Genetic Diversity of *Echinococcus granulosus* Isolated from Humans: A Comparative Study in Two Cystic Echinococcosis Endemic Areas, Turkey and Iran

**DOI:** 10.1155/2020/3054195

**Published:** 2020-04-27

**Authors:** Afshin Barazesh, Bahador Sarkari, Saeed Shahabi, Ahmed Galip Halidi, Abdurrahman Ekici, Selahattin Aydemir, Mahmoud Mahami-Oskouei

**Affiliations:** ^1^Department of Parasitology and Mycology, School of Medicine, Shiraz University of Medical Sciences, Shiraz, Iran; ^2^Department of Microbiology and Parasitology, Faculty of Medicine, Bushehr University of Medical Sciences, Bushehr, Iran; ^3^Basic Sciences in Infectious Diseases Research Center, Shiraz University of Medical Sciences, Shiraz, Iran; ^4^Mus Alparslan University, Bulanik Vocational High School, Muş, Turkey; ^5^Department of Parasitology, Faculty of Medicine, Van YüzüncüYıl University, Van, Turkey; ^6^Department of Parasitology and Mycology, Faculty of Medicine, Tabriz University of Medical Sciences, Tabriz, Iran

## Abstract

Cystic echinococcosis (CE) is one of the most important zoonotic parasitic diseases caused by the larval stage of *Echinococcus granulosus*. Based on molecular studies and DNA sequencing, *E*. *granulosus* has been classified into 10 different genotypes (G1 to G10). Two neighboring countries, Turkey and Iran, are considered the two main foci of CE in the Middle East. The current study is aimed at examining the genotype diversity of *E*. *granulosus* isolated from human clinical samples in Turkey and Iran. Surgically removed human hydatid cysts were collected from East Azerbaijan and Fars provinces in Iran and Van province in Turkey. After extracting DNA, performing PCR, targeting the *cox1* gene, the PCR products were purified from the gel and were sequenced from both directions. The sequences were aligned and compared, using BioEdit and also the BLAST program of GenBank. The maximum likelihood tree was constructed based on the Tamura-Nei model, using the MEGAX software. Phylogenetic analysis showed that the human isolated samples were classified into two major clades: G1 (from Iran and Turkey) and G3 (5 samples from northwestern Iran and one sample from Turkey). The mean and degree of genetic divergence (K2P) between the two major clades, G1 and G3, were 0.2% and 0.7 ± 0.4%, respectively. The findings of the current study revealed that the sheep strain (G1) and the less important strain G3 have major roles in the transmission cycle of CE in two neighboring countries, Iran and Turkey. Therefore, it is necessary to interpose the life cycle of this parasite and reduce the disease burden in livestock and humans by adopting common regional preventive and control policies.

## 1. Introduction

Cystic echinococcosis (CE) is one of the most important zoonotic parasitic diseases caused by the larval stage of *Echinococcus granulosus* [1, 2]. The adult form of this parasite lives in the intestine of the dogs as the definitive hosts, whereas the intermediate hosts are humans and livestock. A human becomes infective through consumption of vegetables and food contaminated with parasite eggs [3].

The infection is widespread worldwide, and it has been reported from all countries in the Middle East, extensively in Turkey, Iran, and Iraq [4–6]. Annual economic losses of CE due to livestock infection and the monetary burden of human CE are substantial in both Turkey and Iran [7–9]. About 1% of surgeries in medical centers in Iran are due to CE [8]. Seroepidemiological surveys have reported a prevalence rate of 1.2 to 21.4% for hydatid cysts in different areas of Iran [9]. Turkey, like Iran, is located in the CE hyperendemic region, but information on the prevalence of human CE in this country is still limited [10, 11]. The estimated surgical case rate of CE is 0.87–6.6 per 100,000 in Turkey [12].

Several studies have shown that *E*. *granulosus* includes a set of different strains with relatively high genetic diversity [13–15]. In recent molecular studies relying on parasitic mitochondrial DNA sequencing, *E*. *granulosus* has been classified into four main groups consisting of 10 different genotypes: *sensu stricto* (genotypes G1 to G3), *equinus* (G4), *ortleppi* (G5), and *canadensis* (G6 to G10). Apart from G4 genotype, all of other strains have been identified from clinical human cases with the most human cases found worldwide being sheep strain (G1 genotype) [14, 15]. This broad parasite genotype diversity affects various features of the parasite including life cycle and transmission, pathogenicity, and biochemical properties as well as its drug susceptibility. It has also been documented that different strains of *E*. *granulosus* have a tendency to infect specific organs of the body and even each genotype of the parasite may have the propensity to infect a particular intermediate host. Therefore, by identifying the dominant genotypes of *E*. *granulosus* in a given geographical area, proper planning can be done to prevent parasite transmission between intermediate and definitive hosts and to prevent the transmission of the disease to humans [16, 17].

Azerbaijan region and Fars province are located in northwest and south of Iran, respectively. Van province is located in the eastern part of Turkey and in the neighborhood of Iran's Azerbaijan region with cold weather and mountainous climate. These areas have always been considered high-risk areas for hydatid cysts. In our previous study, the genotypes of *E*. *granulosus* isolated from livestock from the two countries were comparatively evaluated [16]. The present study is the continuation of our previous study with the aim of comparing the diversity of *E*. *granulosus* genotypes, isolated from human clinical cases obtained from several regions of Turkey and Iran.

## 2. Materials and Methods

### 2.1. Study Area

**Figure 1:**
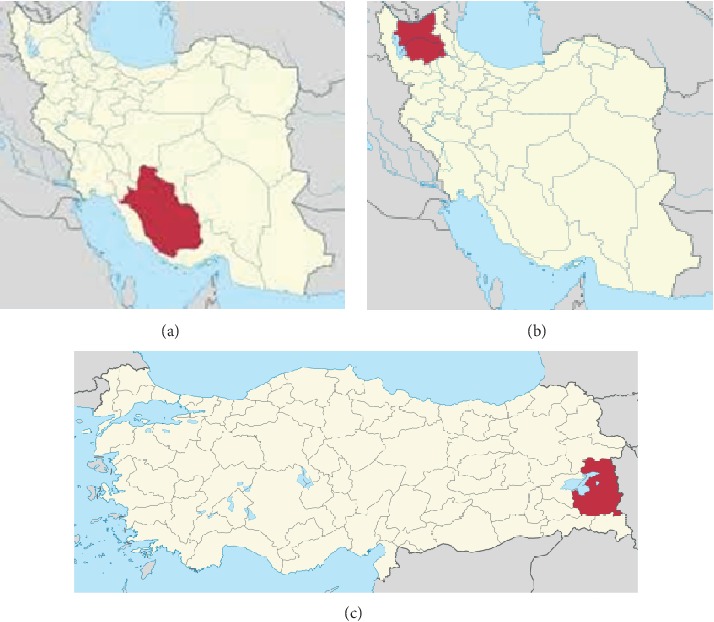
Maps of Iran and Turkey displaying the regions in these two countries where samples were collected. (a) Fars province in Iran, (b) East Azerbaijan province in the northwest of Iran, and (c) Van province in Turkey.

This study was carried out in several different regions of two neighboring countries: Van province in eastern Turkey and East Azerbaijan province in northwestern Iran and Fars province in southern Iran as regions with different climatic conditions (Figure 1).

Azerbaijan region is located in northwestern Iran, and it overlooks the Republic of Azerbaijan and Armenia from the north and to Turkey from the west. The region has cold and mountainous climate with several highlands. Fars province is located in the southern part of Iran with mountainous, temperate, and warm climates. In terms of size and population, Fars is considered the fourth largest and most populous province of Iran. Tabriz and Shiraz metropolises as the capital of East Azerbaijan and Fars provinces are the fourth and fifth most populated cities of Iran, respectively. Van province in eastern Turkey is considered a high-risk area for CE. This area overlooks Lake Van, the largest lake in the Armenian Highlands, from the west and adjoins the Iranian border to the east. Due to its mountainous position and location on the slopes of the Ararat Mountains, it has a cold climate, almost similar to Azerbaijan region.

### 2.2. Sample Preparation

A total of sixty human surgically removed and pathologically confirmed hydatid cysts were collected from Tabriz (capital of East Azerbaijan province) and Shiraz (capital of Fars province) medical centers in Iran and Van province from Turkey (20 samples from each center) and stored in 70% ethanol at -20°C until use.

### 2.3. DNA Extraction from Isolates

The germinal layers, as well as protoscolices of the collected cysts, were used to obtain genomic DNA. With a modification to the procedures of DNA extraction, recommended by a commercial kit manufacturer (Favorgen, Taiwan) and based on the method used in our previous studies [16, 18, 19], DNA was extracted from the samples. Briefly, 100 *μ*L of suspension of parasite protoscolices and 25 mg of germinal layers were prepared in different microtubes. A lysis buffer and proteinase K were added to the sample and incubated for 2 h at 60°C followed by overnight incubation at 37°C. The rest of the procedure was performed according to the kit manufacturer's instructions.

### 2.4. Polymerase Chain Reaction (PCR) and Gel Electrophoresis

The 450 bp fragment of *cox1* from the parasite's mitochondrial DNA was selected as the target gene and amplified, using a pair of highly specific primers, JB3 and JB4.5. The nucleotide sequences of primers and the genomic region of the target gene are shown in Table 1.

The PCR program which was used for the amplification of the genomic fragment consisted of the following: 1X (5′ 95°C) + 40X (45^″^ 94°C + 35^″^ 51°C + 45^″^ 72°C) + 1X (10′ 72°C).

### 2.5. DNA Sequencing

PCR products were electrophoresed on 1.5% agarose gel. PCR products from 40 high-quality samples were cut from the gel and purified, using a commercial kit (TRANS, TransGen Biotech, South Korea), according to the manufacturer's protocol. Finally, 36 PCR products with appropriate quality and purity were sequenced, bilaterally for the *cox1* genomic fragment, using the same pair of primers, used in the PCR assay.

### 2.6. Genetic and Phylogenetic Analyses

The sequences were aligned and compared, using BioEdit and also the BLAST program of GenBank. Moreover, reference sequences for G1-G3 genotypes (G1: KC660075, KF443143, KM100575, and KM513626; G2: AY686559, DQ131582, KM513630, and M84662; and G3: DQ104331, KT074949, HF947568, KU697314, KF443142, JF513060, KF443148, KJ559023, KM513632, and M84663), available in the GenBank, were included in the comparative analysis.

Genetic diversity was measured for the sequences of isolates from Iran (East Azerbaijan and Fars provinces) and Turkey, based on haplotype diversity (Hd) and nucleotide diversity (*π*). Values for the numbers of polymorphic sites, parsimony informative sites, and the average number of nucleotide differences among sequences were estimated. These genetic diversity values were computed by DnaSP software version 5.10 [19].

Phylogenetic relationships were reconstructed, using the maximum parsimony method in MEGAX software, based on the Tamura model. The maximum likelihood (ML) method implemented in PhyML v2.4.4 and Bayesian inference (BI) tree in MrBayes version 3.1.2. The DNA substitution model of TPM1uf+G (−ln*L* = 860.39, *k* = 116, gamma shape = 0.087, *R*(a)[AC] = *R*(f)[GT] = 1, *R*(b)[AG] = *R*(e)[CT] = 11.62, *R*(c)[AT] = *R*(d)[CG] = 2.89) was estimated using the Akaike information criterion using jModelTest version 0.1.1. Bayesian inference was performed with two simultaneous runs and four search chains within each run (three heated chains and one cold chain) for 10,000,000 generations, sampling trees every 1000 generations using the Markov chain Monte Carlo method. The reliability of nodes was assessed using 1000 bootstrap replications for all methods. Trees were rooted with sequences of *E*. *felidis* (accession no. EF558356) and *E*. *multilocularis* (accession no. AB461420). Intraspecific genetic distances were calculated according to the Kimura 2-parameter model by MEGAX software.

## 3. Results

**Figure 2:**
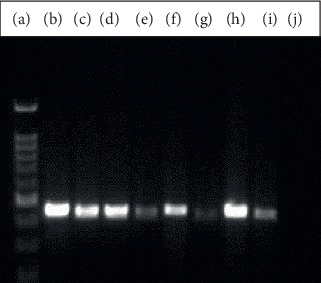
Electrophoresis of PCR products, using JB3 and JB4.5 primers for the *cox1* fragment on 1.5% agarose gel. Lane (a): molecular weight marker, lanes (b)–(h): human isolated samples, lane (i): positive control for *cox1*, and lane (j): negative control.

All of the 60 DNA samples, extracted from the human CE cases, were amplified for the *cox1* genomic fragment.  Figure 2 shows the electrophoretic bands of *cox1* genomic PCR products in a few of the studied samples. From sixty PCR products, 36 samples were sequenced. All 36 sequences were submitted to the GenBank (accession nos. MN807886 to MN807921).

### 3.1. Genetic Structure

There were a total of 412 positions in the final dataset. A total of 9 nucleotide sites were variable, of which three were parsimony-informative. Among these sequences, 8 haplotypes were identified, of which one haplotype with a frequency of 17 was shared between all three regions (Fars, Azerbaijan, and Turkey) and one haplotype with a frequency of six was shared between Turkey and Fars province of Iran. One haplotype with a frequency of six was also shared between East Azerbaijan province of Iran and Van province of Turkey. Overall, haplotype diversity was 0.732 while nucleotide diversity was 0.003. The average number of nucleotide differences (*K*) was 1.39. The group of Turkey sequences (*n* = 9) exhibited 7 haplotypes with haplotype and nucleotide diversities of 0.917 and 0.005, respectively. Four haplotypes were only found in this group. About the sequence group of Fars province (south of Iran, *n* = 7), there were three haplotypes, of which one was shared with Turkey, one was shared with East Azerbaijan, and one haplotype was specific to this group. Haplotype and nucleotide diversities for this group were 0.714 and 0.002, respectively. The last sequence group of East Azerbaijan province of Iran (*n* = 20) exhibited just two haplotypes with haplotype and nucleotide diversities of 0.39 and 0.0019, respectively.

**Figure 3:**
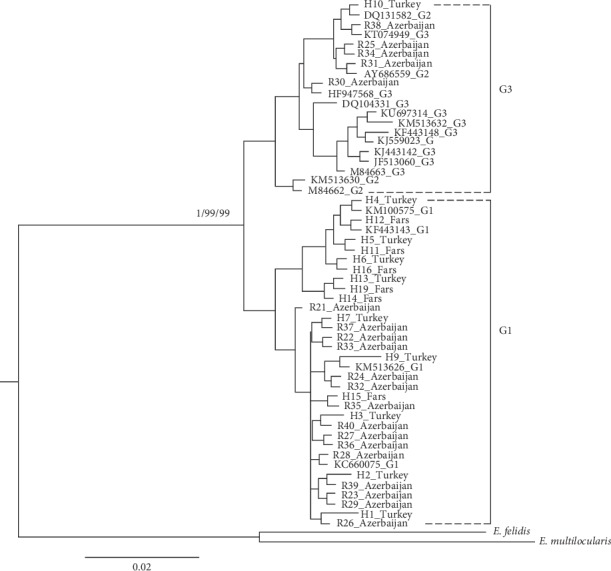
Bayesian 50% majority-rule consensus phylogenetic tree of representative sequences of *Echinococcus granulosus* from Iran and Turkey and reference sequences of other genotypes, using the maximum likelihood method based on the *cox1* gene. Nodal support presented at the node indicates Bayesian posterior probability by Mr. Bayes and bootstrap support for Mp/ML inherence (1000 replicates). Values below 70% are not shown. *E*. *felidis* and *E*. *multilocularis* were used as the outgroup sequence data.

Maximum likelihood (ML), maximum parsimony (MP), and Bayesian analyses produced concordant trees, each revealing that *E*. *granulosus s*. *s*. form a monophyletic lineage concerning selected outgroups. Phylogenetic analysis revealed that the samples were classified into two major clades: G1 (from East Azerbaijan and Fars provinces of Iran and Van province of Turkey) and G3 (5 samples from East Azerbaijan province of Iran and one sample from Van province of Turkey). The monophyly of the clade was strongly supported by BI posterior probability, MP, and ML bootstrap values. The phylogenetic results also demonstrated the reference sequences considered to be G2 (downloaded from GenBank) were grouped in G3 clade. Mean genetic divergence (K2P) among each clade G1 or G3 was 0.2%. The degree of genetic divergence (K2P) between the two major clades G1 and G3 was 0.7 ± 0.4% (Figure 3).

## 4. Discussion

CE has long been considered to be one of the most important health problems throughout the Middle East and has been reported extensively in Iran, Iraq, and Turkey [4, 5]. Currently, CE control is one of WHO's initiatives in these areas [6]. Apart from the enormous economic losses caused by the infection of livestock with hydatid cysts, its human infection also poses serious health risks along with substantial morbidity and even mortality [7–9]. Turkey, like Iran, is located in the hyperendemic region for CE, where significant cases of hydatid cysts are reported annually [11, 12].

Great genetic diversity in the strains of *E*. *granulosus* has been documented, which can affect different parasite characteristics including life cycle, mode of transmission, host specificity, and physiological, biochemical, and parasitic evolution features, as well as pathogenicity and susceptibility to the relevant drugs. These differences would undoubtedly influence the protocols of controlling and preventing this parasitic zoonotic infection. Hence, identifying the different genotypes of *Echinococcus* in any geographical area is justified [16, 17, 20].

In the present study, human isolated CE was collected from different medical centers of Iran and Turkey and the *cox1* genomic fragment from the parasite's mitochondrial DNA was selected as the target gene and amplified, using highly specific primers. All phylogenetic reconstruction methods highly supported the existence of two main strains, G1 and G3, in humans from Iran and Turkey. The results of sequence analysis showed that the sheep strain (G1) was the dominant strain in all evaluated samples and G3 strain was obtained in only 6 samples from both countries (one sample from Van province of Turkey and five samples from East Azerbaijan province of Iran). In the present study, no G3 strain was detected in the samples from Fars province of Iran. This indicates that there were no differences between the samples collected from Fars and East Azerbaijan provinces of Iran and the samples collected from Van province of Turkey in terms of G1 strain dominance. In our recent comparative study conducted on livestock in these areas, the G1 strain was reported to be the predominant one [16]. The results of this study further confirmed the findings of the previous study which indicated that the G1 strain is circulating between humans and livestock in both neighboring countries. With regard to the G3 strain, it was only found in northwestern Iran and the adjacent country of Turkey (one sample). Genetic diversity analysis based on haplotype and nucleotide diversities showed a high genetic diversity (*h* = 0.912) for the parasite isolated from humans from Turkey and less (*h* = 0.39) in humans from East Azerbaijan province of Iran. Considering the close geographical distance between these two regions, this difference between the genetic diversity of the parasite in the two regions is considerable.

In general, *E*. *granulosus sensu stricto* (G1-G3) are the most prevalent strains reported in human hydatid cyst isolates in different CE endemic areas around the world, including Iran [21–24].

In a recent study conducted by Spotin et al. on genetic diversity and population structure of *E*. *granulosus* complex in different geographical regions of Iran, 79 isolates were collected from different hosts (humans, dogs, camels, goats, sheep, and cattle) and examined for genetic diversity, based on the *cox1* genomic fragment. The results showed that the G1 strain was the most common strain among these hosts [25]. In their study, they have found 50 distinct haplotypes among the isolates, which is much higher than the number of haplotypes obtained in the present study. Accordingly, these differences could be due to a diversity of hosts, examined by Spotin et al. In another study performed on several human and cattle CE isolates by targeting the *cox1* genomic fragment in East Azerbaijan province of Iran, all evaluated isolates were identified as sheep genotype (G1), except three human isolates which were classified in G3 genotype [26]. Findings of these studies are consistent with the results of the present study and reaffirm the dominance of G1 in Iran. In a systematic review, Khademvatan et al. evaluated the genotypes of *E*. *granulosus* isolates in different parts of Iran and have introduced G1 as the most dominant genotype in all regions of Iran including northwest (Azerbaijan) and south (Fars). However, a limited number of G3 in Azerbaijan and G6 in Fars were also included in their reports [27].

In a comprehensive study on genetic diversity and population structure of human isolates of *E*. *granulosus* in different parts of Turkey, 84.8% of the isolates were identified as G1 genotype and the remaining 15.2% were G3 genotype [28]. Utuk et al. evaluated the genetic characteristics of hydatid cysts isolated from different intermediate hosts in the eastern and southeastern regions of Turkey, including Van province. Their findings which were based on PCR-RFLP analysis of the ITS1 ribosomal fragment, as well as *cox1* mitochondrial fragment sequencing, showed that the sheep strain (G1) is the most prevalent strain of *E*. *granulosus* in different studied hosts [29]. However, they evaluated only one human isolate and the rest of the cases have been the livestock. In another study by Simsek et al. on CE isolated from cattle and sheep from eastern Turkey, all 54 specimens have been identified in cluster G1-G3, using Single-Stranded Conformation Polymorphism (SSCP) and conventional PCR methods [30].

Molecular studies documented three genotypes (G1–G3) within *E*. *granulosus s*. *s*. based on fragments of the cox1 (366 bp) and nad1 (471 bp) genes [19, 27]. However, G2 is no longer considered a valid genotype as shown in Kinkar et al. (2017) study. Our phylogenetic results, based on the mitochondrial DNA gene (cox1) further confirmed the Kinkar et al. (2017) findings and demonstrated that G2 is not a separate genotype or even a monophyletic cluster [24].

## 5. Conclusion

Findings of the present study revealed that the genetic diversity of *E*. *granulosus sensu stricto* in humans from Turkey was higher than that in Iranian humans isolates, as from 8 identified haplotypes four were specific to the Turkish region. The less genetic diversity of the parasite was found in humans isolates from East Azerbaijan province of Iran. Findings of the study on human CE isolates further revealed that the sheep strain (G1) and the less important G3 strain have major roles in the transmission cycle of hydatid cysts in the two neighboring countries, Iran and Turkey. Therefore, it is necessary to interpose the life cycle of this parasite and reduce the disease burden in livestock and humans by adopting common regional preventive and control policies.

## Figures and Tables

**Table 1:** The characteristics of primers used for amplification of the *cox1* fragment in PCR assay.

Genome	Primers	Sequence
*cox1*	JB3 (F)	5′-TTT TTT GGG CAT CCT GAG GTT TAT-3′
	JB4.5 (R)	5′-TAA AGA AAG AAC ATA ATG AAA ATG-3′

## Data Availability

Data used to support the findings of this study are included in the article.

## References

[1] Thompson R. C. (2017). Biology and systematics of Echinococcus. *Advances in Parasitology*.

[2] Eckert J., Deplazes P. (2004). Biological, epidemiological, and clinical aspects of echinococcosis, a zoonosis of increasing concern. *Clinical Microbiology Reviews*.

[3] Romig T., Deplazes P., Jenkins D. (2017). Chapter Five - Ecology and Life Cycle Patterns of *Echinococcus* Species. *Advances in Parasitology*.

[4] Deplazes P., Rinaldi L., Alvarez Rojas C. A. (2017). Chapter six - global distribution of alveolar and cystic echinococcosis. *Advances in Parasitology*.

[5] Sarkari B., Hosseini F., Abdolahi Khabisi S., Sedaghat F. (2017). Seroprevalence of cystic echinococcosis in blood donors in Fars province, southern Iran. *Parasite Epidemiology and Control*.

[6] World Health O (1988). Echinococcosis/hydatidosis. *Weekly Epidemiological Record= Relevé Épidémiologique Hebdomadaire*.

[7] Dalimi A., Motamedi G. H., Hosseini M. (2002). Echinococcosis/hydatidosis in western Iran. *Veterinary Parasitology*.

[8] Harandi M. F., Budke C. M., Rostami S. (2012). The monetary burden of cystic echinococcosis in Iran. *PLoS Neglected Tropical Diseases*.

[9] Rokni M. B. (2009). Echinococcosis/hydatidosis in Iran. *Iranian Journal of Parasitology*.

[10] Altintaş N. (1998). Cystic and alveolar echinococcosis in Turkey. *Annals of Tropical Medicine & Parasitology*.

[11] Esatgil M. U., Tüzer E. (2007). Prevalence of hydatidosis in slaughtered animals in Thrace, Turkey. *Türkiye Parazitolojii Dergisi*.

[12] Altintas N. (2003). Past to present: echinococcosis in Turkey. *Acta Tropica*.

[13] Sharbatkhori M., Tanzifi A., Rostami S., Rostami M., Harandi M. F. A. S. I. H. I. (2016). *Echinococcus granulosus sensu* lato genotypes in domestic livestock and humans in Golestan province, Iran. *Revista do Instituto de Medicina Tropical de São Paulo*.

[14] Bowles J., Blair D., McManus D. P. (1992). Genetic variants within the genus *Echinococcus* identified by mitochondrial DNA sequencing. *Molecular and Biochemical Parasitology*.

[15] Nakao M., McManus D. P., Schantz P. M., Craig P. S., Ito A. (2006). A molecular phylogeny of the genus *Echinococcus* inferred from complete mitochondrial genomes. *Parasitology*.

[16] Barazesh A., Sarkari B., Sarısu G. (2019). Comparative genotyping of *Echinococcus granulosus* infecting livestock in Turkey and Iran. *Turkish Journal of Parasitology*.

[17] Ahmadi N., Dalimi A. (2006). Characterization of *Echinococcus granulosus* isolates from human, sheep and camel in Iran. *Infection, Genetics and Evolution*.

[18] Barazesh A., Sarkari B., Ebrahimi S., Hami M. (2018). DNA extraction from hydatid cyst protoscolices: comparison of five different methods. *Vet World*.

[19] Davami M. H., Motazedian M. H., Sarkari B. (2010). The changing profile of cutaneous leishmaniasis in a focus of the disease in Jahrom district, southern Iran. *Annals of Tropical Medicine and Parasitology*.

[20] Kheirandish F., Badparva E., Mahmmoudvand H., Beiranvand E., Babaei S., Nasiri B. (2018). Genetic characterization of hydatid cysts isolated from domestic animals in Lorestan province, Western Iran. *Iranian Journal of Parasitology*.

[21] Sarkari B., Mansouri M., Khabisi S. A., Mowlavi G. (2015). Molecular characterization and seroprevalence of *Echinococcus granulosus* in wild boars (*Sus scrofa*) in south-western Iran. *Annals of Parasitology*.

[22] Sarkari B., Fatemie Sfedan A., Moshfe A. (2016). Clinical and molecular evaluation of a case of giant primary splenic hydatid cyst: a case report. *Iranian Journal of Parasitology*.

[23] Alvarez Rojas C. A., Romig T., Lightowlers M. W. (2014). *Echinococcus granulosus sensu* lato genotypes infecting humans – review of current knowledge. *International Journal for Parasitology*.

[24] Kinkar L., Laurimäe T., Acosta-Jamett G. (2018). Distinguishing *Echinococcus granulosus* sensu stricto genotypes G1 and G3 with confidence: a practical guide. *Infection, Genetics and Evolution*.

[25] Spotin A., Mahami-Oskouei M., Harandi M. F. (2017). Genetic variability of *Echinococcus granulosus* complex in various geographical populations of Iran inferred by mitochondrial DNA sequences. *Acta Tropica*.

[26] Mahami-Oskouei M., Ghabouli-Mehrabani N., Miahipour A. (2015). Genotypic characterization of *Echinococcus granulosus* isolates based on the mitochondrial cytochrome c oxidase 1 (cox1) gene in Northwest Iran. *Tropical Biomedicine*.

[27] Khademvatan S., Majidiani H., Foroutan M., Hazrati Tappeh K., Aryamand S., Khalkhali H. R. (2019). *Echinococcus granulosus* genotypes in Iran: a systematic review. *Journal of Helminthology*.

[28] Orsten S., Boufana B., Ciftci T. (2018). Human cystic echinococcosis in Turkey: a preliminary study on DNA polymorphisms of hydatid cysts removed from confirmed patients. *Parasitology Research*.

[29] Utuk A. E., Simsek S., Koroglu E., McManus D. P. (2008). Molecular genetic characterization of different isolates of *Echinococcus granulosus* in east and southeast regions of Turkey. *Acta Tropica*.

[30] Simsek S., Balkaya I., Ciftci A. T., Utuk A. E. (2011). Molecular discrimination of sheep and cattle isolates of *Echinococcus granulosus* by SSCP and conventional PCR in Turkey. *Veterinary Parasitology*.

